# Myeloid-Derived Suppressor Cells Gain Suppressive Function during Neonatal Bacterial Sepsis

**DOI:** 10.3390/ijms22137047

**Published:** 2021-06-30

**Authors:** Jordan K. Vance, Travis W. Rawson, Jessica M. Povroznik, Kathleen M. Brundage, Cory M. Robinson

**Affiliations:** 1 Department of Microbiology, Immunology, and Cell Biology, West Virginia University School of Medicine, Morgantown, WV 26506, USA; jkv0003@mix.wvu.edu (J.K.V.); twr0001@mix.wvu.edu (T.W.R.); Jessica.Povroznik@hsc.wvu.edu (J.M.P.); kbrundage@hsc.wvu.edu (K.M.B.); 2 Vaccine Development Center, Department of Microbiology, Immunology, and Cell Biology, West Virginia University Health Sciences Center, Morgantown, WV 26506, USA

**Keywords:** sepsis, neonatal, myeloid-derived suppressor cells, interleukin-27, toll-like receptor

## Abstract

Neonates are at an increased risk of an infectious disease. This is consistent with an increased abundance of myeloid-derived suppressor cells (MDSCs) compared with older children and adults. Using a murine model of neonatal bacterial sepsis, we demonstrate that MDSCs modulate their activity during an infection to enhance immune suppressive functions. A gene expression analysis shows that MDSCs increased NOS2, Arg-1 and IL-27p28 expression in vitro and in vivo in response to *Escherichia coli* O1:K1:H7 and this is regulated at the level of the gene expression. Changes in the effector gene expression are consistent with increased enzymatic activity and cytokine secretion. The neonatal MDSCs express toll-like receptor (TLR) 2, 4 and 5 capable of recognizing pathogen-associated molecular patterns (PAMPS) on *E. coli*. However, a variable level of effector expression was achieved in response to LPS, peptidoglycan or flagellin. Individual bacterial PAMPs did not stimulate the expression of Arg-l and IL-27p28 equivalently to *E. coli*. However, the upregulation of NOS2 was achieved in response to LPS, peptidoglycan and flagella. The increased immune suppressive profile translated to an enhanced suppression of CD4+ T cell proliferation. Collectively, these findings increase our understanding of the dynamic nature of MDSC activity and suggest that these cells abundant in early life can acquire activity during an infection that suppresses protective immunity.

## 1. Introduction

In 2018, UNICEF reported that 2.5 million infants did not survive the first 28 days of life, with a significant proportion of mortality related to infectious disease [1]. This vulnerability to an infection is consistent with a unique immune profile in neonates relative to adults. Disparities in cytokine production between adult and neonatal innate mononuclear cells have been reported following the bacterial ligand engagement of toll-like receptors (TLR) [2,3,4]. Specifically, previous studies have reported a decreased expression of Th1-associated cytokines interleukin (IL)-12, interferon (IFN)-γ and tumor necrosis factor (TNF)-α in conjunction with an increased production of the Th17-associated cytokines IL-6 and IL-23 in neonatal innate immune cells compared with adults [3,4]. In addition, infants also exhibit increased anti-inflammatory cytokines including IL-10 and IL-27 [3,5,6,7]. Collectively, these cytokine profiles associated with pathogen stimulation support Th17 and Th2-type immunity. Additionally, impaired antigen processing and presentation with consequences on reduced T cell priming have been reported with neonatal cells [8]. While the unique profile of the neonatal immune response maintains a tolerance between the mother and fetus, it renders the neonate inadequate to mount protection against an infection after birth. Mechanistic explanations for a few of the limitations in neonatal immunity and routes to surmount it therapeutically for improved host protection have remained limited. To improve protective responses in neonates, it is imperative to enhance our understanding of the mechanisms that shape early life immunity.

Consistent with an altered immune profile is the abundance of myeloid-derived suppressor cells (MDSCs) early in life [9,10,11]. Independent of gestational age, MDSCs circulate in higher numbers in the umbilical cord blood compared with the peripheral blood of children and adults [9]. MDSC numbers decline abruptly following the neonatal period [10]. Recently, we reported that MDSCs are more abundant in the spleens of neonatal mice than in animals greater than nine days of age [11]. MDSCs are a heterogeneous population of myeloid cells comprised of monocytic (mMDSC, Gr1^+^ CD11b^+^Ly6G^dim/−^Ly6C^+^) and granulocytic (gMDSC, Gr1^+^ CD11b^+^Ly6G^+^Ly6C^dim/−^) subtypes. MDSCs have been characterized in the tumor microenvironment where the cells accumulate and compromise immune surveillance by suppressing T cell activity; however, the MDSC population expands in response to a number of other pathological conditions in humans and mice that include autoimmunity, sepsis and other acute and chronic infections [12,13]. Additionally, recent publications suggest that granulocytic MDSCs may be important regulators of early life immunity as cord blood-derived MDSCs selectively inhibit Th1 responses while promoting Th2 bias and the induction of regulatory T (Treg) cells [14]. MDSCs bias the neonatal immune system toward an anti-inflammatory state that may be suboptimal for pathogen interaction using a variety of mechanisms to suppress immunity [10,12].

Classical MDSC effector mechanisms include the production of nitric oxide (NO), arginase (Arg)-1 activity and the generation of oxidative radicals to inhibit the immune cell function. Our laboratory has previously shown that MDSCs are an important source of the immune suppressive cytokine, IL-27, which is also expressed at elevated levels in neonates [6,7,11]. IL-27 is a pleiotropic cytokine that suppresses inflammatory cytokine production and inflammatory T cell subsets and stimulates IL-10 production by T regulatory cells [15,16]. Elevated levels of early life IL-27 compromise the immune response during neonatal sepsis and promote increased mortality [17]. Macrophages infected with *E. coli* and co-cultured with neonatal MDSCs are impaired in their control of bacteria and the MDSC-mediated suppression of the macrophage function is partially dependent on IL-27 [11]. Thus, IL-27 production should be considered another MDSC mechanism of immune suppression as well as a contributing factor to the immune deficiencies observed in neonates. Changes in MDSC functionality during an acute infection in neonates have not been explored.

In this report, MDSC activity was examined following an infection with *E. coli* O1:K1:H7, a serotype involved with invasive neonatal infections such as sepsis and meningitis [18]. Our results demonstrate that MDSCs increase the expression of nitric oxide synthase (NOS)-2, ARG1 and IL-27 genes during an infection both in vivo and in vitro. This is followed by increased levels of corresponding NOS2 and Arg-1 activity and IL-27 cytokine secretion. Changes in MDSC activity vary in the requirement for exposure to the whole bacterium. Individual bacterial components are not sufficient to drive the same response for ARG1 and IL-27. The observed differences of MDSC functionality during an infection translated to an enhanced immune suppression as infected MDSCs were shown to be more proficient at inhibiting T cell proliferation. Our findings suggest that MDSCs may represent a host-directed therapeutic target to improve the regulation of the neonatal immune response during an acute infection.

## 2. Results

### 2.1. MDSC Effector Function Expression Is Increased during Neonatal Sepsis

**Figure 1:**
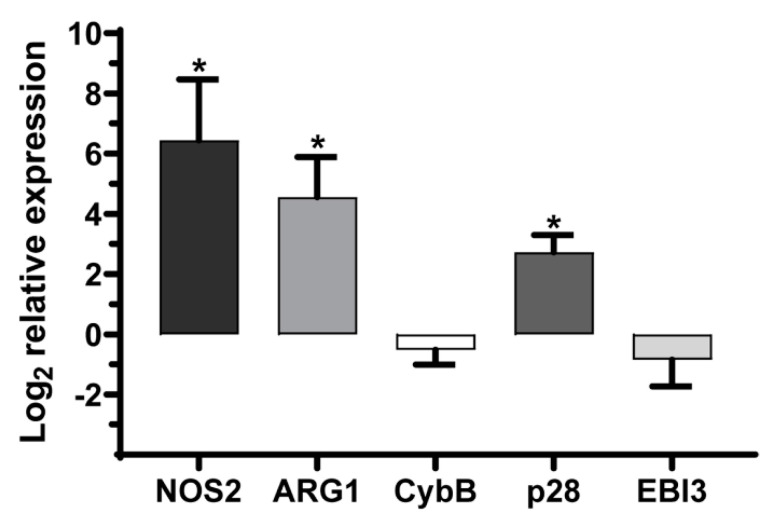
Changes in the MDSC effector gene expression during an infection. C57BL/6 neonatal pups were infected on day 3 or 4 of life with ~10^6^ CFUs. At 18–24 h post-infection, the spleens were harvested from the infected or control mice and MDSCs were isolated by an immunomagnetic selection. RNA was isolated and the expression of nitric oxide synthase (NOS)2, arginase (ARG)1, catalytic subunit of the NADPH oxidase complex (CybB), interleukin (IL)-27p28 and Epstein-Barr virus induced 3 (EBI3) genes was measured by quantitative real time PCR. The values were normalized to β-actin and expressed as a log_2_ change in expression relative to the uninfected control using the formula 2^−ΔΔCt^. The mean results ± standard error from four experiments for NOS2 and three experiments for ARG1, CybB, p28 and EBI3 are shown. An asterisk indicates a statistical significance in the 95% confidence interval relative to the uninfected control that was determined using a paired sample *t*-test.

We previously established a gram-negative model of neonatal sepsis with *E. coli* O1:K1:H7 [17]. This serotype is highly associated with invasive neonatal infections such as sepsis and meningitis [18,19]. In this model, the first day of an infection is a critical period for the control of the systemic bacterial burden. Neonatal pups that are viable on day 2 post-infection most frequently progress to survive and clear the infection [17]. Therefore, to investigate MDSC activity during a critical window of an infection, monocytic and granulocytic subtypes were isolated via an immunomagnetic separation from the spleens of control and infected pups 18–24 h post-infection. This isolation strategy consistently yielded a homogenous population of MDSCs enriched for the granulocytic subtype as shown in Supporting Figure 1 and described previously [11]. RNA was harvested and the effector genes were measured by quantitative PCR. The expression of NOS2, ARG1 and IL-27p28 genes increased significantly in MDSCs isolated from septic mice (Figure 1). The catalytic subunit of the NADPH oxidase complex, CybB was unchanged during the infection (Figure 1). While there was also no significant increase in the expression of the IL-27 subunit EBI3 during the infection, the gene was well expressed in MDSCs from the control and infected neonates. This aligned with the p28 expression serving as the rate-limiting step in the assembly of the functional IL-27 heterodimer.

### 2.2. Direct Exposure to E. coli Drives Changes in Effector Gene Expression

**Figure 2:**
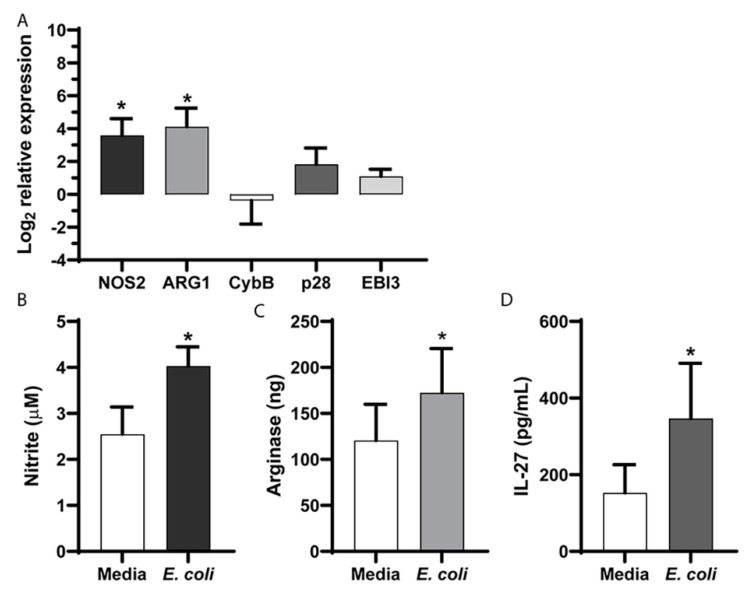
MDSC change in function is directly regulated by bacteria. MDSCs were isolated from the spleens of neonatal naïve mice by an immunomagnetic selection and cultured with *E. coli* at a target MOI of 10 for 30 min prior to the addition of gentamicin (100 µg/mL). (**A**) RNA was isolated at 18–24 h post-infection and the expression of nitric oxide synthase (NOS)2, arginase (ARG)1, catalytic subunit of the NADPH oxidase complex (CybB), interleukin (IL)-27p28 and Epstein-Barr virus induced 3 (EBI3) genes was measured by quantitative real time PCR. The values were normalized to β-actin and expressed as a log_2_ change in expression relative to the uninfected controls using the formula 2^−ΔΔCt^. The mean results ± standard error from five to six experiments are shown. An asterisk indicates a statistical significance in the 95% confidence interval that was determined using a paired sample *t*-test. (**B**) Nitrite levels in the supernatants of the control or infected MDSCs were quantified as described in the Methods section. The mean nitrite levels ± standard error from four experiments are shown. (**C**) Arginase activity was measured from the cell lysates of MDSCs as described in the Methods section. The mean arginase levels ± standard error from six experiments are shown. (**D**) IL-27 concentrations in the supernatants of the control or infected MDSCs were measured by an ELISA. The mean concentrations ± standard error from three experiments are shown. An asterisk indicates a statistical significance in the 95% confidence interval that was determined using a Wilcoxon matched-pairs rank test.

To determine if changes in MDSC effector functions resulted from a direct exposure to bacteria or other signals in the host during an infection, MDSCs were cultured with *E. coli* in vitro. MDSCs were isolated from naïve control pups using an immunomagnetic separation, cultured with *E. coli* for 30 min prior to treatment with gentamicin and then incubated for an additional 18–20 h. A gene expression analysis demonstrated findings similar to those observed in MDSCs from infected neonatal pups. The NOS2 and ARG1 gene expressions were significantly increased following an exposure to bacteria (Figure 2A). Although the increase in the p28 gene expression did not achieve a statistical significance, the pattern of change was similar to that observed in MDSCs from infected pups relative to the controls (Figure 2A). Changes in the gene expression were further confirmed at the level of the protein expression or enzymatic activity. Functional assays demonstrated increased NO production, Arg-1 activity and IL-27 secretion in vitro following a culture with *E. coli* (Figure 2B–D). These findings suggest that MDSC interactions with bacteria alone were sufficient to drive a change in the effector function that was regulated at the gene expression level.

### 2.3. Purified Bacterial Components Drive Differential Changes in the Expression of MDSC Effector Functions Compared with Whole Bacteria

**Figure 3:**
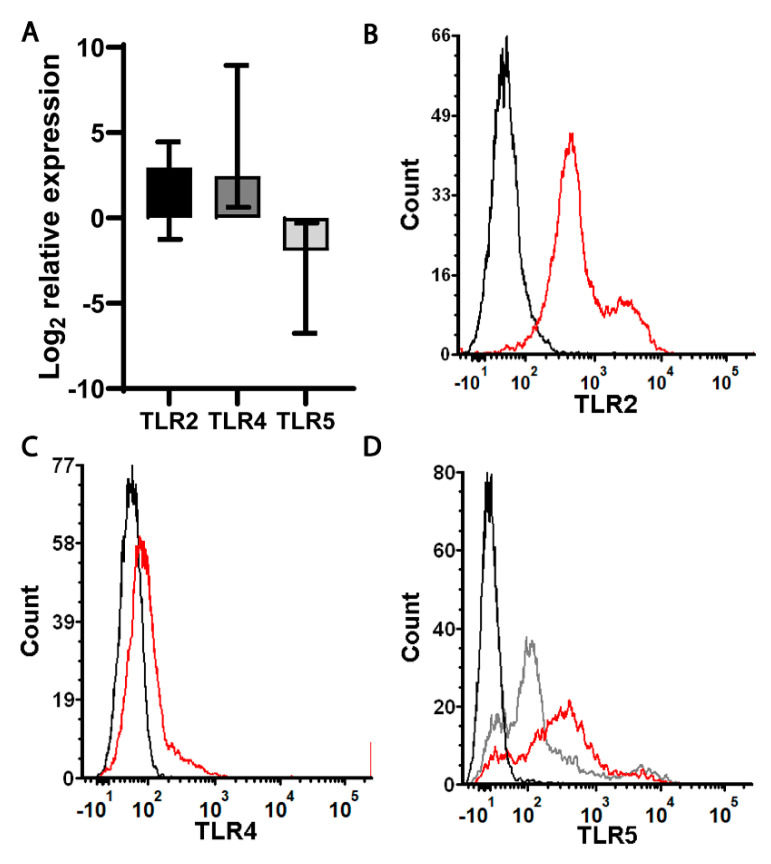
MDSC expression of toll-like receptors (TLRs) in the presence or absence of an infection. (**A**) MDSCs were isolated from the spleens of neonatal naïve mice by an immunomagnetic selection and cultured with *E. coli* at a target MOI of 10 for 30 min prior to the addition of gentamicin (100 µg/mL). RNA was isolated at 18–24 h post-infection and the expression of TLR2, TLR4 and TLR5 genes was measured by quantitative real time PCR. The values were normalized to β-actin, expressed as a log_2_ change relative to the uninfected controls using the formula 2^−ΔΔCt^. The median expression levels with the interquartile range from three to four experiments are shown. (**B**–**D**) The surface expression of TLR2, TLR4 and TLR5 on neonatal MDSCs at a steady state was measured by immunolabeling and flow cytometry. The flow plots are representative of three independent experiments for TLR2, TLR4 and TLR5. The analysis was performed using FCS Express software, version 6.

A direct response to *E. coli* in vitro suggested the involvement of a sensory system to detect bacteria. As such, we hypothesized that the mechanism for effector function changes could involve signaling through pattern recognition receptors to detect bacteria. Therefore, we first confirmed the expression of toll-like receptors (TLRs) that would be predicted to recognize pathogen-associated molecular patterns (PAMPs) specific to *E. coli*. MDSCs were infected in vitro or left untreated as a control. The expression of TLRs predicted to interact with *E. coli*-associated PAMPs were measured by quantitative PCR. This analysis demonstrated that TLR2 and TLR4 were expressed by MDSCs at a baseline and only modestly increased in expression following the stimulation with *E. coli*; however, these changes were not consistent and did not achieve a statistical significance (Figure 3A). TLR5 was expressed by both untreated and *E. coli*-stimulated MDSCs; however, there was a net decrease at the gene expression level that also did not achieve a statistical significance. To confirm the expression of these TLRs at the cell surface, immunolabeling and flow cytometry was performed. This analysis demonstrated that MDSCs derived from neonatal mice showed that TLR2 and TLR5 were well expressed (Figure 3B,D). Surprisingly, while the MDSC population expressed TLR4, it was not robust as evidenced by the shift in fluorescence with the specific antibody relative to the unstained control (Figure 3C).

**Figure 4:**
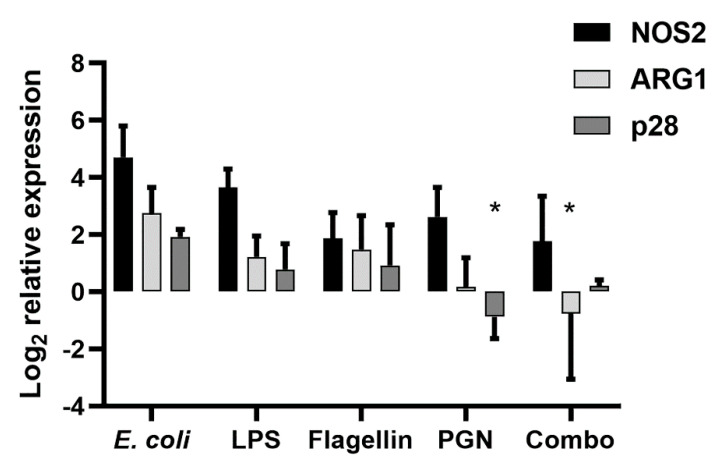
Bacterial components elicit a variable stimulation of the effector gene expression compared with *E. coli*. MDSCs were isolated from the spleens of neonatal control mice by an immunomagnetic selection and cultured with *E. coli* at a target MOI of 10 for 30 min prior to the addition of gentamicin (100 µg/mL) or stimulated with lipopolysaccharide (LPS) (1 µg/mL), flagellin (100 ng/mL), peptidoglycan (PGN) (10 µg/mL), or a cocktail of each component at the described concentrations (combo). RNA was isolated 18–24 h post-infection or stimulation and the expression of NOS2, Arg-1 and IL-27p28 genes was measured by quantitative real time PCR. The values were normalized to β-actin and expressed as a log_2_ change relative to the uninfected controls using the formula 2^−ΔΔCt^. The mean expression ± standard error from seven experiments for individual bacterial components and three experiments for the combination of components are shown. An asterisk indicates a statistical significance in the 95% confidence interval that was determined using a two-way mixed model analysis of variance. Asterisks indicate a statistically significant difference in the expression between individual or the combination of components compared with *E. coli* within a gene.

Peptidoglycan and other lipoproteins from a variety of bacteria are known ligands for TLR2, while LPS is recognized by TLR4 and bacterial flagella interact with TLR5. As the TLR expression profile in Figure 3 indicated that neonatal MDSCs were equipped to respond to bacterial ligands, we explored whether any individual bacterial component and cognate TLR was sufficient to mediate changes in the MDSC functionality. Neonatal MDSCs were stimulated with *E. coli* or purified bacterial components for 18–20 h and RNA was extracted for an analysis of the effector gene (NOS2, ARG1 and p28) expression. A differential response was observed across the effector genes in response to individual bacterial components. Both LPS and peptidoglycan were able to stimulate an equivalent level of the NOS2 gene expression compared with *E. coli* (Figure 4). This response was more intermediate with purified flagella stimulation but not statistically different from *E. coli* (Figure 4). The level of the ARG1 gene expression achieved in response to *E. coli* was only partially induced by any individual component with LPS and peptidoglycan as stronger activators (Figure 4). In contrast, the IL-27p28 expression was not induced by any individual bacterial component (Figure 4). Collectively, these findings suggest that while a few increases in the effector functionality can be achieved in response to bacterial components/TLR ligands, the full complement of the effector functionality requires an activation by an intact bacterial cell. Even the combination of all bacterial components/TLR ligands simultaneously fails to drive the expression of arginase-1 and IL-27p28 comparably with *E. coli*. When the bacterium interacts with the MDSC, several TLRs may be engaged by multiple ligands or cross-linking of the same TLR by a repeated ligand on the surface of the bacterium may be required to generate a signal sufficient to drive the functional changes within the MDSC.

### 2.4. MDSCs from an Infectious Environment More Potently Suppress T Cell Proliferation

**Figure 5:**
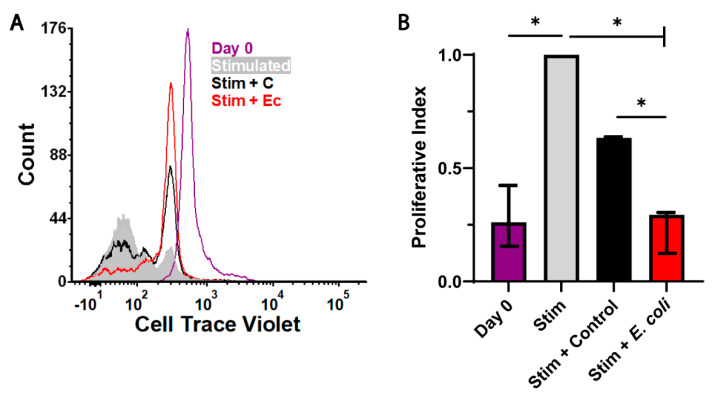
MDSCs isolated from infected neonatal mice exhibit an increased suppression of T cell proliferation. (**A**) CD4+ T cells from adult mice were labeled with Cell Trace Violet and cultured with Dynabeads for four days. Stimulated T cells were cultured ± MDSCs from the control or *E. coli*-infected pups for four days. The T cell proliferation was measured by flow cytometry. An experiment representative of four performed separately is shown. Grey line = day 0 control; grey shaded region = stimulated control; black line = stimulation + control MDSCs; red line = stimulation + MDSCs from infected pups. (**B**) FCS Express (version 6) was used to determine the proliferative index. The median proliferation index + interquartile range for three experiments is shown. An asterisk indicates a statistical significance in the 95% confidence interval that was determined for the indicated comparison using a Mann-Whitney U test.

MDSCs are defined by their ability to limit T cell proliferation specifically via the NOS2 expression and by starving proliferative T cells of available arginine [20,21]. NOS2 is able to oxidize arginine, contributing to a depletion in the environment as well as inhibiting JAK3/STAT5 signaling required for IL-2 receptor upregulation [21]. Similarly, IL-27 has been shown to promote immunosuppressive activity from T cells [22]. Thus, we hypothesized that the observed increase of the effector functions during an infection would translate to an enhanced ability to restrict T cell proliferation by MDSCs from infected neonates. To test this hypothesis, MDSCs were isolated from the spleens of uninfected control and *E. coli*-infected neonatal pups using an immunomagnetic separation. CD4+ T cells were isolated from adult mouse spleens to eliminate the possibility of limitations in the T cell responsiveness associated with neonates, labeled with Cell Trace Violet and expanded by the stimulation of CD3 and CD28 alone or during co-culture at a 1:1 ratio with control or infected MDSCs for four days. An analysis by flow cytometry demonstrated that MDSCs from infected neonates were able to suppress T cell proliferation to a greater level compared with uninfected counterparts (Figure 5). The T cell proliferation reduced by 40% in the presence of control neonatal MDSCs was further reduced by approximately 75% in the presence of MDSCs derived from septic neonates (Figure 5B). This demonstrated that the changes in MDSC activity during the infection and in response to *E. coli* manifested as a heightened level of suppressive activity.

## 3. Discussion

The role of MDSCs in regulating immunity during an acute infection has been understudied. MDSCs are more abundant in neonates and this population has an enhanced susceptibility to an infection [9]. As such, it is important to characterize MDSC activity during an acute infection and the subsequent regulation of the immune response to determine if MDSCs may contribute to the susceptibility to an infection. Here, we have shown that MDSCs are not static and change their functionality in response to *E. coli* O1:K1:H7, an important serotype pathogenic to neonates. This suggested that MDSCs could be key regulators of the immune system during neonatal bacterial sepsis and other acute bacterial infections. MDSCs increased the expression of NOS2, ARG1 and IL-27p28 genes with increased levels of corresponding protein and enzymatic activity when exposed to *E. coli* both in vivo and in vitro. Thus, the functional response to *E. coli* was regulated at the transcriptional level.

To determine which bacterial signals MDSCs may sense and respond to, the cells were stimulated in vitro with purified bacterial components. This included the pathogen-associated molecular pattern molecules LPS, peptidoglycan and flagellin that are represented in *E. coli*. Generally, MDSCs responded in a less robust manner than with whole *E. coli*. The exception was the expression of NOS2 that could be stimulated at a level comparable with that observed in response to *E. coli* with LPS or peptidoglycan. Although changes in the effector gene expression were observed in response to individual components, they were less consistent and did not achieve a statistical significance. Several possible explanations exist. The whole bacterium may be required to generate a strength of signal necessary to induce a functional change in the MDSC. Consistent with this idea, we did not observe a significant change in the TLR gene expression following the stimulation with *E. coli*; this may be necessary for the complete enhanced suppressive function in response to individual bacterial components. Increased avidity may be achieved with bacterial ligands presented in the context of the complete bacterium. It is also possible that the phenotypic change in MDSCs requires the whole bacterium with all of the PAMPs present simultaneously. Rieber et al. reported a dose-responsive induction of human granulocytic MDSCs from a population of PBMCs in response to increasing concentrations of flagellin purified from *Pseudomonas aeruginosa* that was comparable with that observed in response to the whole bacterium [23]. This was consistent with the MDSC expression of TLR5 and in agreement with the requirement of MyD88 for MDSC expansion during polymicrobial sepsis [23,24]. Unlike our study, they did not evaluate the individual activity of MDSCs induced by *P. aeruginosa* or flagellin relative to those not exposed to bacteria. In as much as we are aware, only one other report describes a change in MDSC function during an infection. Brudecki et al. studied adult MDSCs during early and late sepsis using an adult mouse model of cecal ligation and puncture (CLP) [25]. This study showed that the adoptive transfer of MDSCs from early and late sepsis produced differential effects on the immune response. Mice that were adoptively transferred MDSCs from early sepsis displayed an increased inflammatory cytokine production and a reduced recovery of bacteria from the peritoneal cavity but a lower survival. Our findings in the context of early onset neonatal bacterial sepsis established that MDSCs from infected animals had an increased potential to oppose protective immunity. The infected MDSCs harvested from mice 18–24 h post-infection displayed enhanced immune suppressive functions that were consistent with the disease outcomes. However, additional studies are needed to directly connect the enhanced suppressive MDSC function in vivo. Such studies are compounded by a lack of approaches to selectively deplete or inhibit MDSCs without also influencing other granulocyte populations such as neutrophils required for the host response to an acute bacterial infection.

We reported that the increase in the MDSC effector function activity level translated to an increase in the total suppressive activity. MDSCs from infected animals were able to suppress T cell proliferation more than MDSCs from uninfected controls, demonstrating that MDSCs enhanced the immune suppression in response to *E. coli*. TLR engagement ultimately culminates on a different pathway than that used by MDSCs to produce ROS. TLRs use a MyD88 signal transduction independent of the assembly of the NADPH oxidase complex, which leads to ROS expression [26]. As these two signaling mechanisms are different, this could explain why the gene expression of CybB was unchanged in our results. Additionally, an increased ROS may not require a de novo gene expression.

Our findings were consistent with a recent report that described the negative influence of IL-27 on protective immunity and the clearance of bacteria during neonatal sepsis [17]. Neonatal pups deficient in the IL-27 receptor exhibited improved control of bacteria, reduced bacterial-driven inflammation and decreased mortality [17]. In this report, Gr-1+ cells increased the production of IL-27 during an infection. Although this population was not shown specifically to be suppressive MDSCs, it is possible that MDSCs accounted for a considerable portion of the increased IL-27. That would be consistent with the results shown here. IL-27 signaling was not required for the enhanced expression of NOS2 and Arg-1 in MDSCs from infected neonates (data not shown) but IL-27 did have implications in the bacterial clearance and protective immunity. An increased IL-27 production by MDSCs during an infection would be predicted to compromise of a bacterial clearance and increase mortality.

One shortcoming of this work is that we were not able to analyze the gMDSC and mMDSC subtypes separately. MDSCs, as mentioned before, are heterogeneous meaning that there could be differences within the population and their interactions with pathogens. The two subpopulations are found localized together in tissues, thus both the gMDSC and mMDSC populations were analyzed together at the ratios they are found in vivo. However, each subset could exhibit characteristics exclusive to one or the other. As we previously established, the monocytic subtype is significantly outnumbered by the granulocytic subtype. As such, we were not able to isolate a sufficient number of mMDSCs from neonatal mice to perform rigorous studies. For these reasons, we chose to study the collective population to understand how MDSCs can regulate immunity in their natural abundance.

It is important to understand the deficiencies in the development of the immune system and the potential influences on early life susceptibility to an infection and mortality. Strategies to target MDSC abundance or activity that increases during an infection may have potential as host-targeted therapies. We have provided here mechanistic insights into how MDSCs modulate their suppressive activity and compromise the T cell function following a bacterial exposure. This may also have an important implication in the post-sepsis immune suppression for infants that recover from an infection. Thus, the identification of differences in MDSC activity between healthy controls and diseased animals enhances our understanding of early immunity, disease susceptibility and potential host-targeted interventions in this vulnerable population.

## 4. Materials and Methods

### 4.1. Animal Care and Use

Breeding pairs of C57BL/6 mice were purchased from Jackson Laboratories (Bar Harbor, ME) and maintained at West Virginia University School of Medicine. The mice were defined as neonates through eight days of life [6,11]. Both male and female neonates were used for the experimental infections. Blood and tissues were obtained humanely at the indicated time points. All procedures (protocol no. 1708008935) were approved by the West Virginia University Institutional Animal Care and Use Committee on 22 September 2017, renewed on 21 September 2020, and conducted in accordance with the recommendations from the Guide for the Care and Use of Laboratory Animals by the National Research Council (NRC, 2011).

### 4.2. Murine Sepsis Infection Model

*E. coli* O1:K1:H7 (ATCC, Manassas, VA, USA) was prepared with a target inoculum of 2 × 10^6^ bacteria per mouse from pre-titered frozen cultures. Bacteria were washed with phosphate-buffered saline (PBS), centrifuged at 2000× *g* for 5 min at 4 °C and resuspended in the appropriate volume to administer 50 µL per mouse. The mice were infected on day 3 or 4 of life. The litter sizes were approximately 7–8 pups. The bacteria were inoculated subcutaneously into the subscapular region using a 28-gauge insulin needle (Covidien, Dublin, Ireland). The mice were monitored twice daily for signs of morbidity and endpoint criteria. The mice were weighed prior to the infection and 18–24 h later before euthanasia. At the terminal point for each experiment, the mice were euthanized and blood was collected in tubes containing 5 μL of 500 mM ethylenediamine tetraacetate acid (EDTA, Fisher Scientific, Fair Lawn, NJ, USA). The spleens were pooled together in 2 mL of PBS supplemented with 10% fetal bovine serum (FBS, Gemini Bio-Products, West Sacramento, CA, USA) for the isolation of MDSCs.

### 4.3. MDSC Isolation

The spleens were crushed in a 40 µM strainer (Greiner Bio-One, Monroe, NC, USA) within a cell culture suspension dish (Corning, Corning, NY, USA). MDSCs were isolated from single cell suspensions using the murine MDSC Isolation Kit (Miltenyi Biotec, Bergisch Gladbach, Germany) following the manufacturer’s protocol. Immunolabeled cells were sorted using an automated Miltenyi Biotec autoMACS Pro Separator. All experiments incorporated pooled monocytic and granulocytic MDSCs. The purity of the cell population was routinely > 80% Gr-1 and CD11b-positive (Figure S1).

### 4.4. In Vitro Infection and Stimulation

MDSCs were seeded at 2–5 × 10^5^ cells per well in 48-well dishes in Dulbecco’s Modified Eagle Medium supplemented with 10 mM HEPES, 2 mM L-glutamine and 10% fetal bovine serum and infected with *E. coli* O1:K1:H7 at a multiplicity of infection (MOI) of 10. In a few experiments, MDSCs were cultured with purified TLR agonists in parallel at the following concentrations: LPS (MilliporeSigma, St. Louis, MO, USA) at 1 µg/mL; flagellin (Abcam, Cambridge, MA, USA) at 100 ng/mL; peptidoglycan (MilliporeSigma, St. Louis, MO, USA) at 10 µg/mL. The cells were incubated with stimuli at 37 °C with 5% CO_2_ for 30 min and then gentamicin (100 µg/mL) was added for the remainder of the culture.

### 4.5. Quantitative Real Time PCR

MDSCs were harvested with TriReagent^®^ (MilliporeSigma, Burlington, MA, USA) for RNA isolation. Chloroform-based RNA extraction was performed using the Omega BioTek E.Z.N.A.^®^ MicroElute Total RNA Kit (Norcross, GA, USA). RNA was converted into first strand cDNA using the iScript™ cDNA Synthesis Kit (BioRad, Hercules, CA, USA) according to manufacturer’s instructions. The real time cycling of the reactions that included cDNA diluted 2–3-fold, gene-specific primer probe sets (Applied Biosystems, Foster City, CA, USA) and iQ™ Supermix (BioRad) was performed in triplicate using a Step One Plus (Applied Biosytems) real time detection system. For the TLR gene expression analysis, the real time cycling of the reactions that included cDNA diluted 3-fold, 500 nM forward and reverse primers and SsoFast™ EvaGreen^®^ Supermix (BioRad) was performed. The primers used for the TLR expression were manufactured by IDT (Coralville, IA, USA). The specific primer information is outlined in Table 1. The gene-specific amplification was normalized to β-actin and expressed as a log_2_ change relative to the unstimulated control using the formula 2^−ΔΔCt^.

### 4.6. Detection of Reactive Nitrogen

The supernatants from the control or infected MDSCs in vivo or cultured in vitro were removed from the cells and centrifuged at 500× *g* for 5 min to pellet the non-adherent cells. The amount of nitrite present in the supernatant was analyzed using the Measure-IT™ High-Sensitivity Nitrite Assay Kit (Invitrogen, Carlsbad, CA, USA). The supernatant samples (10 µL) or nitrite standards were assayed in triplicate following the manufacturer’s protocol. The fluorescence was quantified from the samples at an excitation/emission of 365/450 nm using a SpectraMax iD3 (Molecular Devices, San Jose, CA, USA).

### 4.7. Arginase Activity Assay

The arginase activity was measured using a protocol described previously [11]. A whole cell extract of MDSCs (2–5 × 10^5^ cells) was prepared using a RIPA lysis buffer (Santa Cruz Biotechnology, Dallas, TX, USA) supplemented with PMSF (2 mM) and a protease inhibitor cocktail (diluted 1:100, Santa Cruz Biotechnology) on ice. For a few experiments, MDSCs were first stimulated with *E. coli* O1:K1:H7 or purified TLR ligands as described above for 18–20 h. The lysate was clarified by centrifuging 10,000× *g* for 10 min at 4 °C. To assess the amount of arginase activity, the cell lysate (25 µL) or recombinant human arginase (R&D Systems, Minneapolis, MN, USA) was combined with 0.1 mM 5,5-dithio-bis-(2-nitrobenzoic acid) (DTNB, Sigma-Aldrich, St. Louis, MO, USA), 0.9 mM thioarginine (Cayman Chemical, Ann Arbor, MI, USA) and PBS to a final working volume of 100 µL and incubated for 30 min at 37 °C with 5% CO_2_. The absorbance was measured at 412 nm with a SpectraMax iD3 (Molecular Devices, San Jose, CA, USA).

### 4.8. Interleukin-27 Secretion

The IL-27 heterodimer concentration was measured in the culture supernatants using the Legend Max Mouse IL-27 ELISA Kit (Biolegend, San Diego, CA, USA) following the manufacturer’s protocol. The undiluted samples and standards were assayed in duplicate and in parallel.

### 4.9. T Cell Proliferation Assay

Single cell suspensions were prepared from adult C57BL/6 mouse spleens as described above. The T cells were isolated from single cell suspensions using the CD4 isolation kit (Miltenyi Biotec, Bergisch Gladbach, Germany) following manufacturer protocol. The isolated CD4+ T cells were stained with Cell Trace Violet (Invitrogen, Carlsbad, CA, USA) in the dark for twenty minutes at 37 °C after which the cells were washed extensively to remove excess dye. For the Time 0 sample, an aliquot of cells was fixed prior to culturing with MDSCs. MDSCs were isolated from the neonatal spleens as described above using immunomagnetic labeling. CD4+ T cells were seeded at 2 × 10^5^ T cells per well in a non-tissue culture-treated 96-well plate. The cells were stimulated with Dynabeads^®^ Mouse T-Activator CD3/CD28 (Thermofisher, Waltham, MA, USA) in a 1:1 ratio and co-cultured with MDSCs derived from either the control or *E. coli*-infected mice. After four days, the cells were separated from the Dynabeads^®^, pelleted at 300× *g* for 10 min at 4 °C and placed in PBS that contained 0.4% paraformaldehyde.

### 4.10. Flow Cytometry

MDSCs isolated from the neonatal spleens as described were labeled for a phenotypic analysis by flow cytometry with 0.5 µg of antibody per 2 × 10^5^ cells. Gr1 PE, Ly6C PE and Ly6G FITC were obtained from EBioscience (San Diego, CA, USA). CDllb APC-Vio770, TLR2 PE and TLR4 APC were obtained from Miltenyi Biotec (Bergisch Gladbach, Germany). The TLR5 antibody (Proteintech, Rosemont, IL, USA) was used to stain the total splenocytes prior to MDSC isolation for 1 h on ice in the dark and washed twice (300× *g*, 10 min, 4 °C). The primary antibody was revealed with goat anti-mouse IgG (H+L) cross-adsorbed with the secondary antibody, Alexa Fluor 568 (Thermofisher, Waltham, MA, USA), for 45 min on ice in the dark, washed and then subsequently stained for the MDSC isolation. After staining and separation, the cells were subsequently placed in PBS that contained 0.4% paraformaldehyde. The data were acquired using a BD-LSRFortessa (Becton Dickinson, Franklin Lakes, NJ, USA) and FACS Diva version 8.0 software. A minimum of 10,000 events were collected per sample. The data files were analyzed using FCS Express Software (version 6) (De Novo Software, Los Angeles, CA, USA).

### 4.11. Blocking and Neutralization Assay

MDSCs were isolated as described above and seeded at 3–5 × 10^5^ per well in a 96-well cell culture plate (Corning, Corning, NY, USA). The cells were subsequently treated in the presence or absence of TLR2, TLR4, (Miltenyi, Bergisch, Germany) or TLR5 (Invivogen, San Diego, CA, USA) neutralizing antibody (0.3–0.5 µg per 3–5 × 10^5^ cells) for 45 min and incubated at 37 °C with 5% CO_2_ for 18–20 h. After the neutralization, MDSCs were stimulated with *E. coli* at an MOI of 10 for 30 min before treatment with 100 µg/mL of gentamicin. The cells were harvested with TriReagent (MilliporeSigma, Burlington, MA, USA) for the RNA isolation and the gene expression analysis.

## Figures and Tables

**Table 1:** Detailed primer information for those used in the gene expression studies.

Target Gene	Manufacturer	Commercial Product Number or Sequence
Beta-actin (ActB)	ThermoFisher	Mm01205647_g1
Nitric oxide synthase (NOS2)	ThermoFisher	Mm00440488_m1
Arginase-1 (Arg-1)	ThermoFisher	Mm01190441_g1
NADPH-oxidase complex (CybB)	ThermoFisher	Mm00627011_m1
Interleukin-27 subunit p28 (IL-27p28)	ThermoFisher	Mm00461164_m1
Epstein–Barr Induced-3 (EBI3)	ThermoFisher	Mm00469294_m1
Toll-like receptor 2 (TLR2)	IDT	F-5′-ACCGAAACCTCAGACAAAGC-3′ R-5′-CATCACATGACAGAGACTCCTG-3′
Toll-like receptor 4 (TLR4)	IDT	F-5′-TTCAGAACTTCAGTGGCTGG-3′ R-5′-TGTT AGTCCAGAGAAACTTCCTG-3′
Toll-like receptor 5 (TLR5)	IDT	F-5′-CATCTGTGAGACACCCCTTG-3′ R-5′-CAGGGAGATA TTACCAACACGG-3′

## Data Availability

The data presented in this study are available on request from the corresponding author.

## Supplementary Materials

The following are available online at https://www.mdpi.com/article/10.3390/ijms22137047/s1.
