# Supplementary material for: Myeloid-Derived Suppressor Cells Gain Suppressive Function during Neonatal Bacterial Sepsis
Source: Int J Mol Sci. 2021 Jun 30;22(13):7047. doi: 10.3390/ijms22137047 (PMC8268718; doi:10.3390/ijms22137047)
Supplement: Supplementary file 1 [file ijms-22-07047-s001.zip › ijms-1282491-supplementary.pdf]

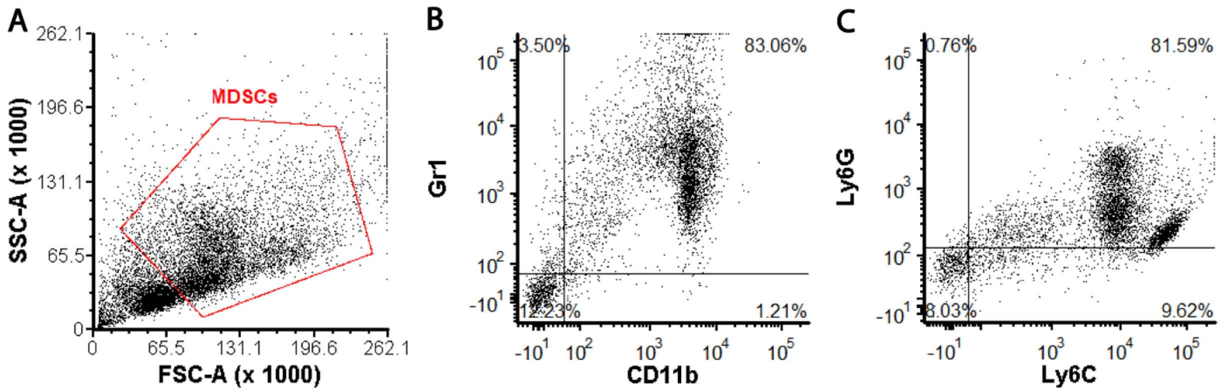

Supplementary Figure S1. *MDSC purity and phenotype.* Neonatal MDSCs were immunolabeled for the indicated marker and analyzed by flow cytometry. (A) Dot plot representing the gated area for cell analysis. CD11b/Gr1 (B) and Ly6C/Ly6G (C) specific labeling was used to demonstrate cellular phenotype.
